# Prospective evaluation of plasma levels of ANGPT2, TuM2PK, and VEGF in patients with renal cell carcinoma

**DOI:** 10.1186/s12894-015-0019-4

**Published:** 2015-04-03

**Authors:** Bishoy A Gayed, Jessica Gillen, Alana Christie, Samuel Peña-Llopis, Xian-Jin Xie, Jingsheng Yan, Jose A Karam, Ganesh Raj, Arthur I Sagalowsky, Yair Lotan, Vitaly Margulis, James Brugarolas

**Affiliations:** Department of Urology, University of Texas Southwestern Medical Center, Dallas, Texas USA; Department Internal Medicine, University of Texas Southwestern Medical Center, Dallas, Texas USA; Department of Clinical Science, University of Texas Southwestern Medical Center, Dallas, Texas USA; Department of Developmental Biology, University of Texas Southwestern Medical Center, Dallas, Texas USA; Department of Urology, MD Anderson Cancer Center, Houston, Texas USA; Department of Urology, UT Southwestern Medical Center at Dallas, 5323 Harry Hines Blvd., Dallas, 75390-9110 Texas USA

**Keywords:** Biomarkers, Angiogenesis, Prospective, Renal cell carcinoma, Tumor metabolism

## Abstract

**Background:**

To assess pathological correlations and temporal trends of Angiopoietin-2 (ANGPT2), vascular endothelial growth factor (VEGF) and M2 Pyruvate kinase (TuM2PK), markers of tumor vascular development and metabolism, in patients with renal cell carcinoma (RCC).

**Methods:**

We prospectively collected plasma samples from 89 patients who underwent surgical/ablative therapy for RCC and 38 patients with benign disease (nephrolithiasis, hematuria without apparent neoplastic origin, or renal cysts). In RCC patients, marker levels were compared between at least 1 preoperative and 1 postoperative time point generally 3 weeks after surgery. Marker temporal trends were assessed using the Wilcoxon sign-rank test. Plasma VEGF, ANGPT2, and TuM2PK levels were determined by ELISA and tested for association with pathological variables.

**Results:**

Median age was comparable between groups. 83/89 (93%) of the cohort underwent surgical extirpation. 82% of the tumors were organ confined (T ≤2, N0). Only ANGPT2 exhibited significantly elevated preoperative levels in patients with RCC compared to benign disease (*p* = 0.046). Elevated preoperative levels of ANGPT2 and TuM2PK significantly correlated with increased tumor size and advanced grade (*p* < 0.05). Chromophobe RCC exhibited higher levels of ANGPT2 compared to other histologies (*p* < 0.05). A decline in marker level after surgery was not observed, likely due to the timing of the analyses.

**Conclusion:**

Our results suggest that ANGPT2 is a marker of RCC. Additionally, ANGPT2 and TuM2PK significantly correlated with several adverse pathological features. Further studies are needed to determine clinical applicability.

## Background

In 2014, 63920 new diagnoses, and 13860 deaths attributed to tumors of the kidney and renal pelvis are expected [1]. 5-year cancer specific survival (CSS) probability rates for patients with localized and locally advanced disease are around 80-90% and 20-50%, respectively [2]. Advances in surgical techniques and the development of targeted therapies have lead to improved oncologic outcomes of patients with RCC, however, survival of patients with advanced disease continues to be deficient [3]. A better understanding of the biology of tumors is required to improve oncological outcomes.

Central to the development of RCC of clear-cell type (ccRCC) is the loss of VHL with activation of a hypoxia-adaptive program that involves metabolic changes and angiogenesis. Our understanding of the nature of ccRCC has led to the development of targeted agents that antagonize VEGF signaling [4]. Currently, therapies target the VEGF ligand or its receptor. Other determinants of angiogenesis are being investigated, including Angiopoietin 2 (ANGPT2) [5,6]. ANGPT2 is found at sites of vascular remodeling and functions by undermining vascular foundation [7].

Loss of VHL induces profound metabolic changes. For instance, we recently showed that VHL inactivation in the mouse is sufficient to inhibit mitochondrial respiration [8]. Tumor cells often rely on aerobic glycolysis for energy generation, which makes carbon sources available for anabolic processes. One protein that plays a critical role in tumor metabolism is pyruvate kinase. Several isoforms of this enzyme exist, however, the M2 isoform (M2PK) is specifically implicated in oncogenesis, and is overexpressed in tumor cells [9]. Studies have shown that the dimeric form (TuM2PK) may be a marker of malignant renal disease [10]. In addition, TuM2PK may be a useful predictor of recurrence in patients with RCC [11]. However, the current role of TuM2PK continues to be undefined.

Currently, prognostic factors such as stage and grade fail to incorporate the individual biological heterogeneity and clinical behavior of RCC [12]. Thus, there is a strong impetus for detecting and incorporating biomarkers into clinical practice that expose the biological behavior of tumors and aid in risk assessment [13].

In this prospective feasibility study, we analyzed a panel of potential RCC markers (VEGF, TuM2PK, and ANGPT2) in patients with RCC vs. a control group with benign renal disease. We correlated the levels of the marker with pathologic features of the tumor at surgery and evaluated the levels postoperatively.

## Methods

### Patient selection

Between October 2008 and March 2010, patients presenting to the UT Southwestern Medical Center Urology Clinic with a renal mass suspicious for RCC as well those with presumed benign etiology were enrolled in an UT Southwestern Medical Center IRB approved tissue and blood repository protocol. Patients enrolled in the study signed written consent. Research was carried out in compliance with the Helsinki Declaration. 125 patients were followed from the time of diagnosis to at least 2 preoperative time points and 1 postoperative time point taken more than 24 h after surgery. Of these, 9 presented with metastatic disease and 3 patients developed other malignancies, and were withdrawn, leaving 113 patients (102 underwent surgical nephrectomy and 11 radiofrequency ablation). Benign pathology was reported in 13 surgical patients and 5 additional patients were withdrawn because were not left NED after surgery. 5 patients treated with ablative intervention had either no biopsy, were benign, or insufficient material was available. After applying these criteria, 90 patients qualified for this analysis and 89 underwent ELISA assays. 38 patients qualified as controls with either of the following benign conditions: nephrolithiasis, hematuria of presumed benign etiology, or simple renal cysts. Computerized tomography (CT) scans were used to evaluate urologic conditions and establish radiologic absence of malignancy. Further, patients with hematuria also underwent complete workup, including cytology, imaging, and cystoscopy to rule out malignancy.

### Collection and storage of samples

Peripheral venous blood was collected from patients with RCC at preoperative and postoperative time points. Patients serving as controls had blood drawn at their initial clinic visit. Blood was collected in EDTA tubes, and centrifuged, typically within 15 minutes. Plasma samples were aliquoted and stored at −80°C until analysis.

### Elisa assays

Plasma from each pre and postoperative time point was evaluated. Serum samples, collected for other purposes, were not used for this study as platelet degranulation during clotting may lead to falsely elevated levels of the marker [14]. Plasma VEGF and ANGPT2 levels were determined by ELISA according to the manufacturer’s instructions (R & D Systems, Minneapolis, MN, USA). Plasma TuM2PK levels were also determined by ELISA according to the manufacturer’s instructions (ScheBo, Wettenberg, Germany). Each time point was run in duplicate and all samples for each patient were run on the same plate. Standards and a set of controls were run on each plate. ELISA results for each marker were displayed as heatmaps by normalizing the values of each patient to the number of standard deviations above or below the average.

### Statistics

Patient characteristics are displayed using medians, ranges, frequencies, and percentages. Where applicable, marker levels were calculated as the patient’s average preoperative draw and the average postoperative draw. To evaluate marker trends over time the Wilcoxon sign-ranks test was used. Wilcoxon rank sum test was used to find if there was a difference between RCC patients and control patients. All statistics were performed using software from GraphPad Prism version 5.03 (GraphPad Software, San Diego, CA, USA) and SAS version 9.3 (SAS Institute Inc., Cary, NC, USA). A *p*-value < 0.05 was considered significant. False discovery rate control was used for the p-values from the testing between marker levels and pathological variables.

## Results

### Clinical features

Table 1 outlines the demographic and clinical characteristics of both cohorts. A total of 127 patients were included in the study, 89 patients underwent treatment of their renal mass and 38 patients presented with benign conditions that served as the control group. The two groups were comparable with respect to age, gender, and race. 83/89 (93%) patients with renal masses underwent extirpative resection, with either partial or radical nephrectomy. 82% of the tumors were organ confined (pT ≤2, N0) and 78% had clear cell histology.

**Table 1:** **Characteristics of RCC and control patients**

**Variable**	**RCC (** ***n*** **= 89)**	**Controls (** ***n*** **= 38)**
Age Median (range)	62 (25-85)	57 (23-89)
Sex - no. (%)		
Male	52 (58.4)	20 (52.6)
Female	37 (41.6)	18 (47.4)
Race - no. (%)		
Caucasian	66 (74.2)	28 (70.0)
African American	9 (10.1)	6 (15.0)
Hispanic	9 (10.1)	6 (15.0)
East Indian	4 (4.5)	0
Asian	1 (1.1)	0
Diagnosis - no. (%)		
RCC	89 (100)	
Stones		27 (71.1)
Hematuria		9 (23.7)
Renal Cyst		2 (5.3)
Approach - no. (%)		
Ablation	6 (6.7)	
Nephrectomy	83 (93.3)	
Open	42 (50.6)	
Laparoscopic	41 (49.4)	
Radical	36 (43.4)	
Partial	47 (56.6)	
AJCC Stage† - no. (%)		
I	60 (73.2)	
II	7 (8.5)	
III	15 (18.3)	
IV	0	
pT Classification† - no. (%)		
T1a	41 (49.4)	
T1b	20 (24.1)	
T2	7 (8.4)	
T3	1 (1.2)	
T3a	5 (6.0)	
T3b	9 (10.8)	
T3c	0	
T4	0	
Pathologic Size† - median (range)	4.1 (1.3-25)	
LN Involvement† - no. (%)		
NX	71 (85.5)	
N0	11 (13.3)	
N1	1 (1.2)	
Histology - no. (%)		
Clear Cell	69 (77.5)	
Papillary	14 (15.7)	
Chromophobe	5 (5.6)	
Unclassified RCC	1 (1.1)	
Fuhrman Grade† - no. (%)		
1	10 (12.0)	
2	44 (53.01)	
3	26 (31.33)	
4	3 (3.6)	

^†^Analysis does not include patients treated with RFA.

### Association of marker levels with malignancy

**Figure 1:**
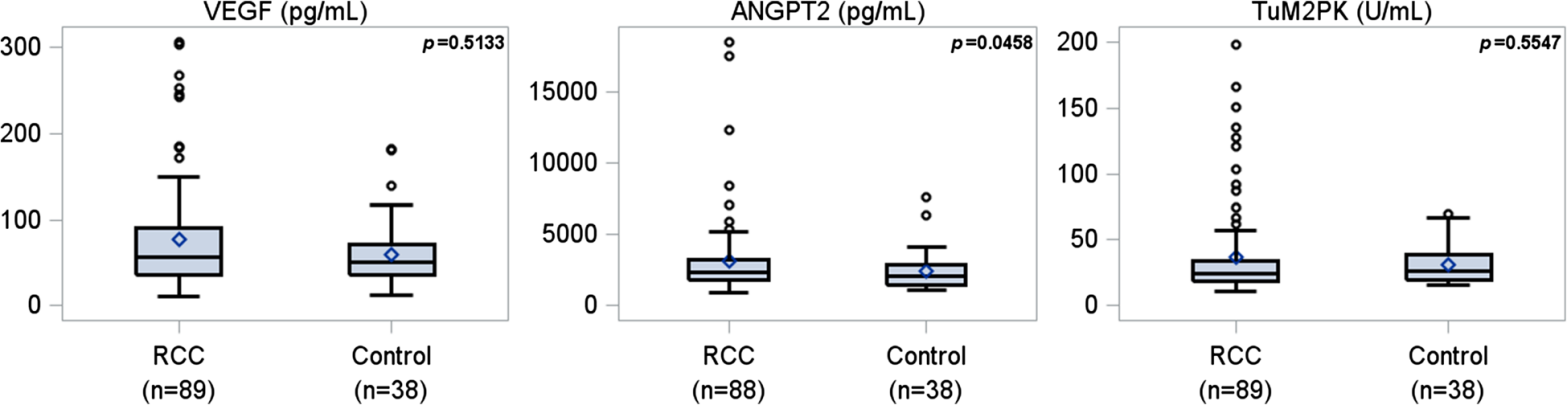
**Wilcoxon rank sum test for differences in marker levels for 1st preoperative time point versus controls.**

Median time between the operation and the last postoperative draw was 25 days (interquartile range 18–187 days). ANGPT2 exhibited significantly elevated preoperative levels in patients with RCC (*p* = 0.046) compared to those with benign disease, while preoperative TuM2PK and VEGF levels were comparable between patients with benign and malignant disease (Figure 1).

### Temporal changes of markers

**Figure 2:**
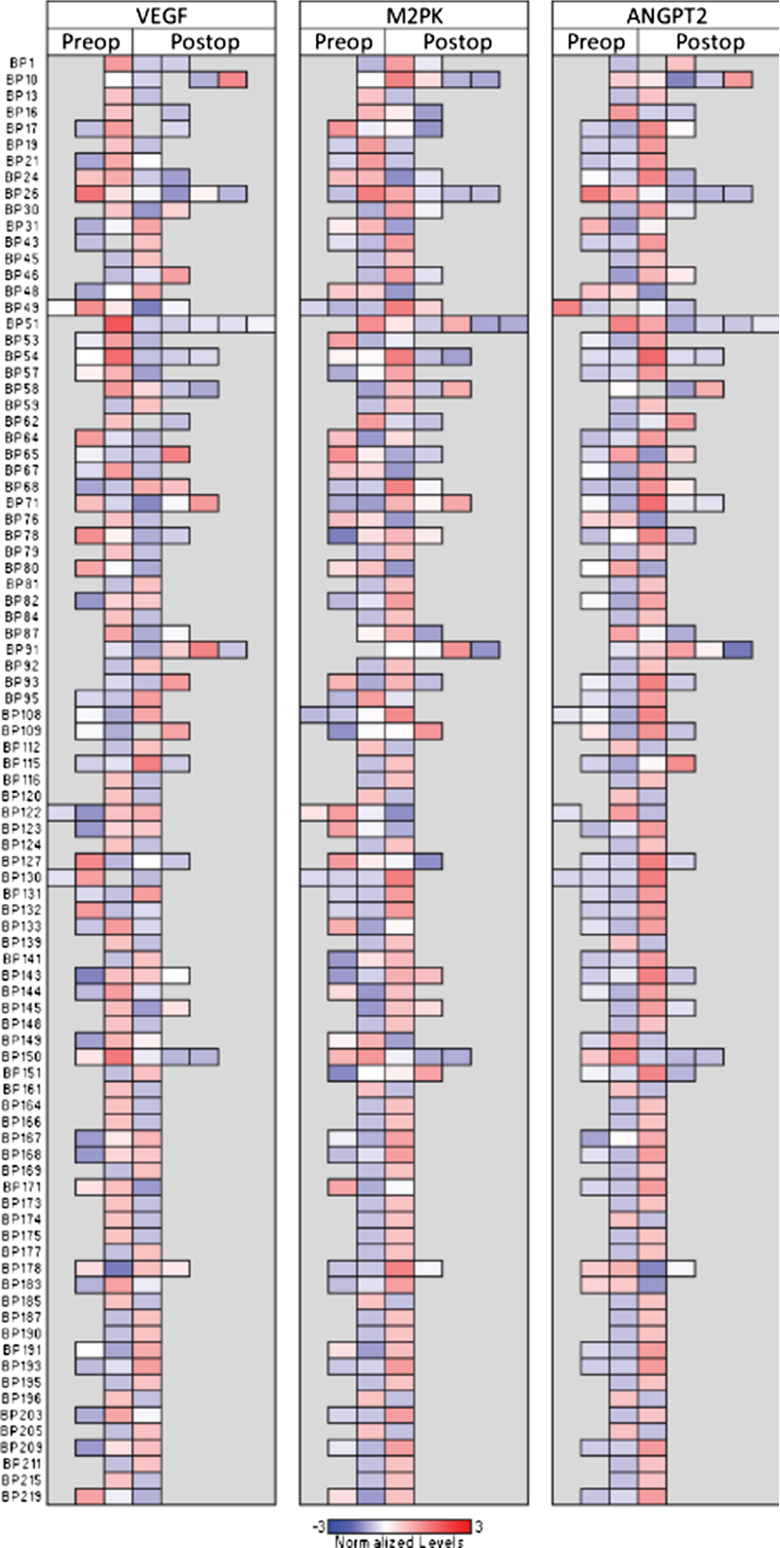
**Heatmap representation of VEGF, M2PK, and ANGPT2 plasma levels by patient, over time.** For each marker and patient, values were normalized to the number of standard deviations above (red) of below (blue) the average. Samples are arranged in chronological order for each patient. The number of samples available for each patient varies, and isolated gray boxes for a particular patient represent missing values for the particular analysis.

Figure 2 is a heatmap representation of the different markers for each patient over time.

Table 2 shows how marker levels are affected over time. We calculated the median difference of 1st postoperative levels vs. the average of all the preoperative levels (which we perceived to be most representative of preoperative levels). Our results showed that ANGPT2 and TuM2PK levels increased significantly. Similar results were observed when all postoperative samples were included in the analysis.

**Table 2:** **Wilcoxon signed rank test for median of difference in marker levels between pairs of time points**

	**1st post – Avg. Preoperative**			**Avg. all but 1** ^**st**^ **post – Avg. preoperative**			**Avg. all post – Avg. preoperative**			**Avg. all but 1** ^**st**^ **Post – 1** ^**st**^ **postoperative**		
	***n***	**Median**	***p***	***n***	**Median**	***p***	***n***	**Median**	***p***	***n***	**Median**	***p***
ANGPT2	88	318.1	0.0004	26	2.4	0.3914	88	253.1	0.0082	26	−300.2	<0.0001
M2PK	89	13.0	<0.0001	27	−2.1	0.2880	89	12.4	<0.0001	27	−8.6	0.0003
VEGF	89	−6.1	0.2021	23	−2.2	0.1973	89	−4.0	0.3581	23	4.0	0.6799

When the first postoperative sample was excluded (despite being ~3 weeks after the surgery), the differences largely disappeared. In keeping with this finding, there was a significant difference in ANGPT2 and TuM2PK levels between the first and remaining postop values. Overall, these data are consistent with the notion that surgery induces plasma ANGPT2 and TuM2PK levels.

### Association of pathological features with markers levels

**Figure 3:**
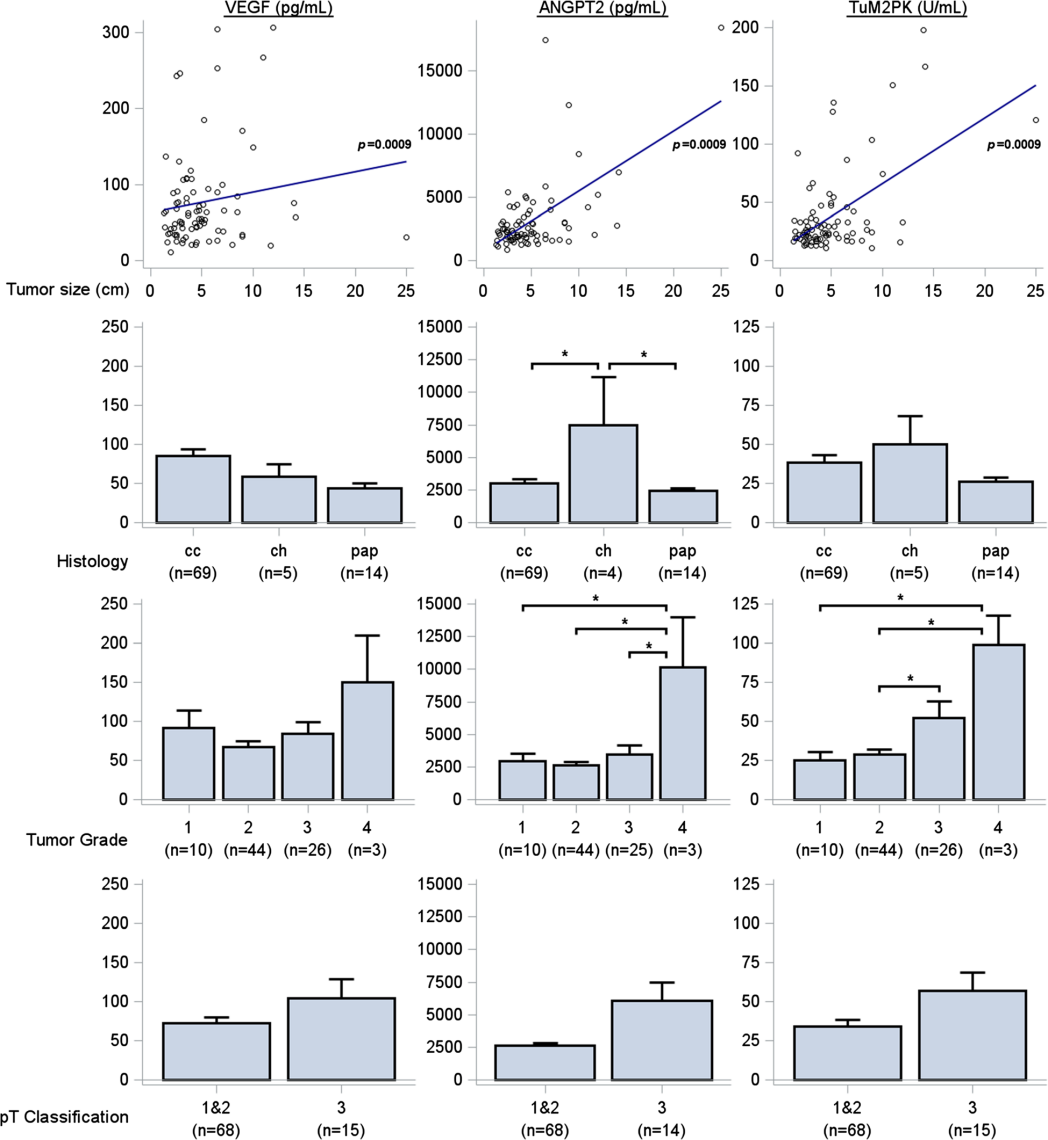
**Pathological features and association with marker levels.**

Interestingly, elevated preoperative levels of ANGPT2 and TuM2PK significantly correlated with several adverse pathological features (Figure 3). There was a significant correlation between ANGPT2 levels and tumor size (*p* = 0.0009). Similarly, TuM2PK levels were also correlated with tumor size (*p* = 0.0009). In addition, there was a correlation between ANGPT2 and TuM2PK and grade. Higher levels of both ANGPT2 and TuM2PK were observed in grade 4 tumors (*p* < 0.05). In addition, chromophobe RCC exhibited significantly higher levels of ANGPT2 compared to other histologies (*p* < 0.05) (Figure 3). No correlation was seen between VEGF levels and adverse pathological features.

## Discussion

Circulating tumor biomarkers may assist with primary diagnosis, determination of recurrence and prognosis. However, no suitable renal cancer biomarkers have been identified and incorporated in clinical practice. In this study, we explored a sensitive paradigm. We evaluated samples from patients with a primary in place and performed comparisons of circulating proteins before and after surgery. Intra-patient comparisons are likely to minimize confounding by other variables. In addition, we compared biomarker levels between renal cancer patients and a group of controls with non-malignant disease. By enrolling surgical candidates, we were able to correlate circulating biomarker levels to the pathological features of the tumor. We focused our studies on VEGF, ANGPT2 and TuM2PK.

One pathway that is being extensively studied is the angiopoietin/Tie-2 pathway. This pathway is comprised not only of angiopoietin 1 (ANGPT1), but also of ANGPT2, and its receptor vascular receptor tyrosine kinase Tie-2. These factors play a significant role in neovascularization [15,16]. ANGPT1 has been shown to be involved in vascular development, while ANGPT2 functions to undermine vascular integrity [7,15]. ANGPT2 overexpression has also been shown to augment tumor angiogenesis [17]. Sallinen et al. showed that patients with ovarian carcinoma had significantly higher levels of ANGPT2 than individuals with benign disease. Further, elevated ANGPT2 levels correlated with advanced stage as well as worse DFS and OS [18]. Others have shown that ANGPT2 to be a biomarker of disease status, adverse pathological features, and worse oncological outcomes [19,20]. While the definitive role of ANGPT2 in RCC remains undefined, studies exist showing that ANGPT2 concentrations appear to be elevated in patients with RCC [21,22]. Efforts are also ongoing to target angiopoietin/Tie-2 system with drugs such as AMG-386 and CVX-060 in patients with RCC [23].

TuM2PK has been implicated as a driver of aerobic glycolysis, and shown to be a marker of malignancy in several neoplasms [9]. Landt et al. revealed that TuM2PK levels can distinguish between malignant and premalignant cervical lesions. Additionally, they showed that increased levels of TuM2PK were associated with node positive as well as metastatic disease [24]. A recent meta-analysis showed that elevated TuM2PK levels correlated with malignancy as well as extent of disease in patients with GI malignancy [25].

Few reports exist regarding the role of TuM2PK in patients with RCC. Nisman et al. showed that elevated levels of TuM2Pk were significantly associated with worse pathological features, including grade and tumor necrosis [11]. Their results also revealed that patients with elevated circulating TuM2PK had worse 5-year RFS than patients with normal marker levels (55% vs. 94% *p* < 0.001). On multivariate analysis, TuM2PK was an independent predictor of disease recurrence (*p* = 0.04) [11]. In an interesting study by Roigas et al. plasma levels of TuM2PK were compared between healthy patients and patients with RCC. Elevated levels of TuM2PK were significantly elevated in patients with RCC than healthy patients [26]. Conversely, Varga et al. concluded that TuM2PK is not an adequate marker for RCC [27].

In our study, we prospectively analyzed the prognostic significance of 3 markers of angiogenesis and metabolism. Most importantly, elevated preoperative levels of ANGTP2 and TuM2PK were significantly associated with several adverse pathological features, notably size and grade.

While anti-VEGF therapies have become mainstay of treatment for patients with metastatic clear cell RCC, the role and efficacy of targeted therapies in non-clear cell variants remains unclear [28]. Developing therapies based on molecular markers specific for other histologies could improve oncological outcomes. In our study, ANGTP2 levels were significantly higher in patients with chromophobe RCC, than those with either clear cell or papillary RCC, suggesting that ANGPT2/TIE2 system may play a particularly important role in chromophobe tumors and may serve as a target for therapy. However, not all chromophobe samples exhibited the same degree of ANGPT2 elevation.

Disappointingly, temporal trends did not show the predicted decrease in marker levels that we expected. This could be due to several reasons. The short follow-up of the study did not allow for enough time for the marker levels to decrease. Also, the timing of when marker samples were collected could account for the variability in levels. However, currently the timing of when marker samples should be collected remains undefined. Nevertheless, some reports show that it may take 11 weeks for elevated levels of TuM2PK to normalize [10].

The definitive goal of developing novel biomarkers would be incorporation into current prognostic tools. Novel markers, such as ANGPT2 and TuM2PK could improve oncological outcomes in patients with RCC by identifying patients who may benefit from a particular therapy, thus individualizing treatment plans. Biomarker-based scoring algorithms, such as the BioScore, which is based on the expression levels of B7-H1, survivin, and Ki67, help to predict the likelihood of RCC specific death [29]. Individuals with high Bioscores are associated with a higher rate of death from RCC than individuals with low BioScores [29]. Thus, biomarker incorporation into current prognostic models could serve as an excellent risk stratification tool that both individualizes therapy and as well as directs treatment.

Our study has several limitations. The number of patients is modest. While we concluded that elevated marker levels were significantly seen in certain subtypes of RCC, our data is still limited by the number of patients included in the study. As with prospective studies, results are based on availability of samples postoperatively and patient follow up. Despite these challenges, our study does show significant associations between some markers and adverse pathological parameters.

## Conclusion

In our preliminary study, plasma levels of ANGPT2 and TuM2PK obtained prior to ablation or surgery for renal masses, were increased compared to controls and were associated with several aggressive pathological features including tumor size and grade. Our findings support further research into the role of circulating proteins as a means to augment current prognostic predictors of outcome in patients with kidney cancer.

## Abbreviations

ANGPT2: Angiopoietin-2
VEGF: Vascular endothelial growth factor
TuM2PK: M2 pyruvate kinase
RCC: Renal cell carcinoma
CSS: Cancer specific survival
ccRCC: Clear-cell type
